# Welcome to *Children* — A New Open Access Journal Dedicated to Sharing Medical Research Relevant to Children’s Health

**DOI:** 10.3390/children1010001

**Published:** 2013-09-06

**Authors:** Sari Acra

**Affiliations:** Monroe Carell Jr. Children’s Hospital at Vanderbilt University, 2200 Children’s Way, 10233 Doctors’ Office Tower, Nashville, TN 37232, USA; E-Mail: sari.acra@vanderbilt.edu; Tel.: +1-615-343-9034

I am very excited about the upcoming launch of *Children*, a journal dedicated to the streamlined yet scientifically rigorous electronic dissemination of peer-reviewed science related to childhood health and disease in developed and developing countries.

The future of our world depends on the health and well-being of all its children. Thus, the global health issues facing children today will determine medical history. Unfortunately, as the world becomes more of a global information village in many respects, there have remained impediments to eliminating regional disparities in sharing health information, be it in the fields of infectious diseases, nutrition or cancer risks, to name but a few. It is my hope that *Children* will be a forum for sharing information, and engaging in discussions and dialogue relevant to the care of children, unimpeded by limitations imposed by traditional print media. We also hope to dedicate entire issues to timely and relevant single-topic publications.

As the founding Editor-in-Chief of *Children*, I look forward to working with the great talents represented by you, my colleagues in the discipline of Pediatrics, to mold this publication into the premiere scientific journal focused on children’s health issues. I cherish your roles, not only as contributors, but also as expert reviewers. In that effort we will be also supported by a great editorial team assembled by MDPI, who will be a readily available resource for all of us. 

*Children* will focus on sharing clinical, epidemiological, translational and basic science research relevant to children’s health. Moreover, the aim of the journal is to be a vehicle to highlight under-represented pediatric disciplines, to emphasize interdisciplinary research and to disseminate advances in knowledge in global child health. In addition to original research, the journal will publish expert editorials and commentary, clinical case reports, and insightful rapid communications reflecting the latest developments in pediatric medicine. By publishing meritorious articles as soon as the editorial review process is completed, rather than at predefined intervals, *Children* will also permit rapid open-access sharing of new information, allowing us to reach the broadest audience in the most expedient fashion.

It is our hope to publish the first papers in the fall, and I invite you and colleagues at your respective institutions to be among the early contributors to this effort.

Again, thank you for joining me on this wonderful journey of discovery. 

